# Withdrawal from Long-Term Use of Unusually High-Dose Oxazepam

**DOI:** 10.1155/2021/2140723

**Published:** 2021-11-05

**Authors:** Antti Mustonen, Juhani Leijala, Johanna Aronranta, Antero Lassila, Mauri Aalto, Janne Koskimäki

**Affiliations:** ^1^Faculty of Medicine and Health Technology, Tampere University, Tampere, Finland; ^2^Center for Life Course Health Research, University of Oulu, Oulu, Finland; ^3^Department of Psychiatry, Central Hospital of Southern Ostrobothnia, Hanneksenrinne 7, FI-60220 Seinäjoki, Finland; ^4^Neuroscience Center, HiLIFE, University of Helsinki, Box 63, 00014 Helsinki, Finland; ^5^Division of Clinical Neurosciences, Department of Neurosurgery, Turku University Hospital and University of Turku, P.O. Box 52, Hämeentie 11, FI-20521 Turku, Finland

## Abstract

Benzodiazepine (BZD) misuse is a worldwide problem that healthcare professionals encounter in daily practice. High-dose BZD withdrawal is usually a long process that may require referral to an inpatient rehabilitation unit. Relapses after withdrawal are common. BZD withdrawal can cause complications including seizures, suicidal behavior, anxiety, and depression. Guidelines describe tapering protocols for modest doses; however, protocols for exceptionally high-dose BZD withdrawal are not well described. Herein, we describe a BZD tapering protocol for a patient with daily use of high-dose (1800 mg) oxazepam (OXP). The BZD tapering was administered in an inpatient psychiatric hospital, and the outcome was evaluated monthly after discharge for three months. This report describes a unique case of high-dose OXP withdrawal and also outlines an optional protocol to apply when clinicians encounter these unusual cases.

## 1. Background

Benzodiazepine (BZD) medications are among the most commonly prescribed psychotropic medications in the world [1–4]. Short-term use of BZD is considered to be relatively safe, although dependence develops in approximately 39% of patients who use benzodiazepines for longer than 1 month [5]. Furthermore, longitudinal studies with large samples have reported associations between BZD misuse and psychiatric comorbidity including anxiety and mood disorders [5–7].

Withdrawal symptoms are typically psychological, but abrupt discontinuation can lead to severe life-threatening events such as seizures especially in cases of high-dose use [8]. Clinical withdrawal guidelines most commonly describe tapering protocols for moderate BZD doses [9]. However, an exctreme high-dose BZD taper protocol is not well described. According to previous literature, treatment is typically tailored individually and usually requires hospitalization [10–12]. Herein, we present a case of extremely high-dose use of oxazepam (OXP) and a successful tapering protocolin an inpatient setting.

## 2. Case Description

A 60-year-old upper middle class female had history of anxiety and depression for the past 30 years for which she was treated with varying doses of OXP after discontinuation of several SSRI/SNRI medications due to various side effects. After having a recent 5-minute seizure, the patient was referred to the emergency room (ER). Use of extremely high doses of OXP usage was discovered in the ER. According to an electronical national prescription registry, the patient had been on a daily dose of 1800 mg OXP (60 tablets of 30 mg formulation) during the last six months, which was confirmed by the patient. This is equivalent to approximately 600 mg of diazepam [13]. The patient reported minor alcohol use (AUDIT 3 points). In addition, the patient used acetaminophen/codeine combination medication for atypical headache that was prescribed by her neurologist. Patients reported daily doses of 3000 mg of acetaminophen and 180 mg of codeine. However, electronical prescription registry check-up revealed that drug purchaces had more than doubled during the past month. In blood tests, however, only S-GT showed minor increase 99 U/I (normal < 40 U/I), and other hepatic function laboratory tests were within reference values. Increased P-CK (584 U/I; *normal* < 210 U/I) was measured corresponding with recent seizure severity. A short follow-up at the intensive care unit was performed, and thereafter, she was transferred to a psychiatric hospital for further evaluation and treatment.

**Figure 1:**
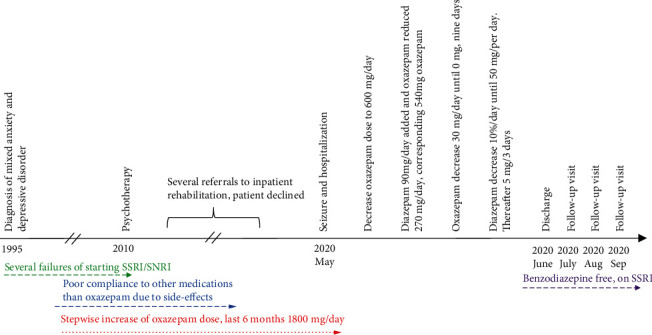
Clinical course of the patient.

At first, OXP dose was reduced to 600 mg per day (four 30 mg tablets five times per day). Acetaminophen/codeine 500/30 mg combination dose was decreased to the standard dose of 2 tablets 3 times a day during the first day of hospitalization with intention of withdrawal in the later stages of her treatment. Levetiracetam 500 mg was started two times a day for seizure prevention (Figure 1). Levomepromazine 50 mg was started to promote sleep. For high blood pressure and prevention of other BZD/opiate withdrawal-related symptoms, the patient received clonidine (*α*-2-agonist) 75 *μ*g three times per day. After the first two days, OXP dose was decreased to 270 mg per day and 90 mg of (30 mg three times a day) diazepam (DZP) was prescribed, equivalent dose corresponding to 540 mg OXP per day. During the first several days, the patient reported minor increase in anxiety. Sleeping disturbances (difficulty to fall asleep) were still present, and levomepromazine dose was increased to 100 mg with favorable response. After DZP 90 mg and OXP 270 mg had been administered for two days, OXP dose was gradually tapered with a decrease of 30 mg in a day. This taper from OXP took a total of nine days, after which the patient remained on 90 mg of DZP per day. Surprisingly, withdrawal symptoms did not worsen, and at the OXP taper endpoint, the patient continued to report only minor anxiety.

We continued to taper off DZP about 10% of the dose per day. However, the patient started to report increased anxiety after dose of 50 mg was achieved and, thus, the pace of withdrawing was slowed down. Hence, DZP dose was reduced 5 mg in every three days. DZP was tapered off within one month without complications and the patient was discharged from the hospital.

On the day of discharge, the patient reported occasional mild anxiety but was sleeping well with reduced levomepromazine dose of 50 mg. Levetiracetam was continued after discharge for two weeks with a dose of 500 mg twice a day which was then was reduced to 500 mg once a day for two weeks. Furthermore, levomepromazine was switched to trazodone 50 mg per day to treat insomnia.

During the hospitalization period, the patient was prescribed sertraline which was later discontinued due to side effects (increased anxiety). This was successfully switched to venlafaxine. One month after discharge, the patient was scheduled an outpatient appointment. The patient had discontinued taking venlafaxine due to side effects (increased anxiety). After discussing potential pros and cons of an additional antidepressant medication, paroxetine was prescribed. During the next two follow-up visits, the patient showed resolution in anxiety. The quality of life of the patient had increased markedly and she was able toenjoy daily life with her family. Relapses with BNZ did not occur during the three month period after discharge. Psychosocial support and other psychiatric treatment continued in the outpatient clinic.

## 3. Discussion

Tapering off very high-dose benzodiazepine was feasible within a relatively short time period. However, the treatment may require hospitalization and close monitoring as abrupt discontinuation of high-dose BZD medication may lead to severe life-threatening complications such as seizures.

The withdrawal from 1800 mg OXP to 50 mg DZP (corresponding to 150 mg OXP) went surprisingly well and was done in a relatively short amount of time without major withdrawal symptoms. Eventual tapering off was also completed with DZP. We decided to use DZP over OXP in this setting due to the longer half-life and more suitable pharmacokinetics to prevent seizures and possible BZD-related withdrawal symptoms including anxiety. Additionally, for seizure prevention, levetiracetam treatment was successfully administered as a preventive medication in this case. Furthermore, clonidine seemed effective in the treatment of high blood pressure and other psychological and somatic symptoms caused by the withdrawal.

This report demonstrates the strength of a nationwide prescription registry as an excellent tool to identify prescription drug misuse. Physicians are advised to check patients' previous prescriptions when prescribing central nervous system drugs to identify misuse of these medications. In this case, however, the patient received all the medications mainly from one physician, and the use of very high-dose OXP use was not noticed until ER.

This presented case report has several limitations. It must be acknowledged that this is a single case and treatment cannot necessarily be generalized. We did not measure OXP concentrations from blood or urine, which may be considered a weakness, and thus, we do not have objective confirmatory data about the use of high-dose OXP. However, this is not a common practice in Finland and would not have a major impact on the clinical decisions in an inpatient psychiatric hospital with close monitoring of the patient. Furthermore, the patient tolerated 600 mg of oxazepam without any sedative effect suggesting unusually high tolerance consistent with the reported dose. In fact, additional sedative medications were required to support the patient's sleep and anxiety (including clonidine and levomepromazine). It is a good practice to monitor the patient's urine samples during withdrawal especially in outpatient settings for abstinence. However, in this case, this was not considered because we monitored national electronical prescription database and also had strong family involvement increasing our confidence regarding abstinence. Furthermore, when considering patient's background, age, and the rural environment she was living in, it was highly unlikely that the patient would have access to benzodiazepines from other sources than doctors.

This case report demonstrates that tapering off extremely high-doses of BZDs is possible within a relatively short time period in hospital setting. In this case we administered antiepileptics, *α*-2-agonists, and long-acting benzodiazepines in treatment. However, we acknowledge that successful withdrawal from very high-doses of BNZ may generally require more time. Maintaining the course of abstinence requires close collaboration of inpatient and outpatient treatment facilities. Physicians should preemptively inform patients about the risks of abrupt discontinuation of high-dose BZD medication, which includes life-threatening complications.

## Data Availability

Due to the nature of this clinical case report, anonymized data is available on request.

## Abbreviations

BZD: Benzodiazepines
DZP: Diazepam
OXP: Oxazepam.

## Additional Points

*Highlights*. (i) Extremely high-dose benzodiazepine tapering may require hospitalization. (ii) Withdrawal may be feasible in a relatively short period of time although close monitoring is necessary. (iii) Abrupt discontinuation of benzodiazepine treatment may lead to known severe life-threatening events such as seizures.
